# Knowledge and Understanding of Personal Protective Equipment Use among Laborer Population of the Nepalese Workforce

**DOI:** 10.1155/2021/7679185

**Published:** 2021-01-28

**Authors:** Pratikshya Gurung, Maginsh Dahal, Kushalata Baral, Ankit Pathak, Sudip Khanal

**Affiliations:** ^1^Department of Public Health, Asian College for Advance Studies, Purbanchal University, Lalitpur, Nepal; ^2^Department of Public Health, Nobel College, Pokhara University, Sinamangal, Kathmandu, Nepal

## Abstract

The constructing laborers are mainly unskilled, untrained, migrant, socially backward, and uneducated with low bargaining power. Thus, we assessed the knowledge and prevalence on occupational safety and health (OSH) of laborers working at private constructing sites. A descriptive cross-sectional study of 229 laborers working at private constructing sites selected by 30 cluster sampling methods from the Lalitpur metropolitan city and Mahalaxmi municipality was conducted using a structured questionnaire and observation checklist. EpiData and SPSS were used for data analysis. Most of the laborers (62%) had inadequate knowledge on OSH. The level of knowledge was significantly associated with sex, education, and family type at 95% CI (*p* value < 0.05). The prevalence of occupational accidents within a year was 19.7% and was significantly associated with the use of Personal Protective Equipment (PPE) at 95% CI (*p* value < 0.05). About one-fifth of the participants had occupational accidents within a year because of the inadequate knowledge of OSH.

## 1. Introduction

According to World Health Organization (WHO), occupational health is a multidisciplinary activity aimed at the protection and promotion of the health of workers by preventing and controlling occupational diseases and accidents and by eliminating occupational factors and conditions hazardous to health and safety at work. Occupational safety is the risk identification at the workplace and preventive measures taken to reduce or eliminate the hazard which may lead to accidents [1].

According to the International Labor Organization (ILO), constructing activities cover a building including excavation and the constructing, structural alteration, renovation, repair, maintenance, and demolition of all types of buildings or structures. It also states that constructing sites are any site at which any of the processes or operations of stated constructing activities are carried on and workers are any person who is engaged in constructing activities [2].

The laborers face many kinds of injury which are superficial injuries and wound, fractures, dislocations, sprains, strains, traumatic amputations, internal injuries, burns, frostbite, chemical burns, acute poisoning, and infections [3]. The unsafe working conditions, lack of supervision and training, use of old machinery and equipment, lack of sufficient maintenance, bad housekeeping practices, violation of safety rules, and overcrowded production units with very congested areas are the causes of occupational hazards as listed in the article of Occupational Safety and Health Studies in Nepal [4].

In addition, constructing sites provide unfriendly working conditions, exposing to one of the toughest environments at a workplace to laborers [5]. Lack of legal provisions and enforcements and adequate awareness in Nepal has led laborers to work in hazardous situation without using proper protection. Furthermore, the construction of apartment buildings is adding more risk for workers as high-rise buildings increase risks like falling of workers and falling of materials upon workers [6]. Also, Personal Protective Equipment (PPE) is not available in most working places and available PPE are also not effectively used due to a low level of awareness [6].

Besides, the possibility of a fatality is five times more likely in constructing than in the manufacturing industry while the risk of a major injury is two and half times higher [7]. It is also estimated that approximately 20,000 workers meet accidents at the workplace which lead to about 200 lives lost in Nepal [8, 9].

Occupational health and safety are important in constructing sites as a large number of laborers work in risky environments. Limited research focused on the occupational safety and health among laborers in Nepal, especially in the Lalitpur metropolitan city and Mahalaxmi municipality. Therefore, findings from this study will help to assess the knowledge on occupational safety and prevalence of use of PPE among laborers working at private constructing sites in Lalitpur district and will address these knowledge gaps and provide baseline data to improve the safety and health of constructing laborers in Nepal.

## 2. Methods

A descriptive cross-sectional study was conducted among laborers working in private building constructing sites of Mahalaxmi Municipality and Lalitpur Metropolitan City. For this study, 30 chowks (an open market area in a city at the junction of two roads) having constructing sites were identified from Lalitpur Metropolitan City and Mahalaxmi Municipality. Each chowk was determined as one cluster and the total sample size was divided into 30 clusters. Each cluster consists of approximately 8-sample size. Instead of a bottle, a pen was rotated standing at the center of each chowk and the path where the cap of the pen ended was selected [10]. The first constructing site that comes across the selected path was taken. Then, in that constructing site, the 8 samples were purposively taken. If 8 samples were not completed in the first constructing site, then, again, the pen rotate method was done to select the second constructing site for the completion of 8 samples from one chowk.

The sample size was determined using Raosoft software. According to a descriptive cross-sectional study on practice related to occupational health and safety among workers of brick factories at Bhaktapur, Nepal, where the level of practice of PPE use was 86.3% [11] which was taken as prevalence in this study to determine sample size. The calculated sample size was 181 with 5% margin of error, 95% CI. With the addition of 10% nonresponse rate, the final sample size was 199. However, the sample size was increased to 229-sample size in order to get accurate results so that it could be generalized for further studies.

The questionnaire and observation list were developed in the local language and researchers having similar backgrounds were requested to provide feedback to make the tools more valid. The questionnaire was also pretested to 30 laborers working in similar settings and the tool showed good internal reliability with a Cronbach's coefficient alpha of *α* = 0.712. The pretested structured questionnaire and observation checklist were used to collect the primary data through face-to-face interview and observation. The checklist depended upon the number of the visited private constructing sites and was filled before leaving the visited constructing sites. The structured questionnaire included questions related to knowledge, PPE use, and occupational accidents and the observation checklist included a checklist related to the PPE use and working condition of constructing sites.

The chowks of Lalitpur Metropolitan City and Mahalaxmi Municipality having at least three private building constructing sites were selected in order to have enough participants from a chowk during the time of data collection. Those laborers who were working in private building constructing sites were included as fewer studies have been done in laborers of private constructing sites and the possibility of a fatality is five times more likely in constructing than in a manufacturing industry. Those laborers who were willing to participate were taken to maintain the ethics of the study. Only the occupational accidents that occurred within a year were taken in this study to reduce the recall bias. The chowks having less than three private building constructing sites were not considered so that there will not be any insufficiency of laborers during data collection. The employees like managers and engineers other than the laborers were not included in this study as this study focuses only on the laborers of private constructing sites.

Knowledge of laborers on OSH is the information on OSH acquired by the laborers through experience and education. A total of 19 questions related to the knowledge of laborers on OSH were prepared which included questions on knowledge of OSH and its importance, first aid, OSH program, OSH training, knowledge on PPE, safety inspection, safety meeting, preventive and control measures for occupational accidents, and OHS policy of Nepal. Each question was given 1 score. For the Yes/No answer question, Yes was given 1 score and No was given 0 score. For a multiple response question, each answer was given score 1. Then, the total score for the multiple response question was calculated and then converted to a score out of 1. For example, if the multiple response question had 4 answers and the participant answered two options out of four answers, then the total score for it will be 2 which will be converted to 0.5 out of 1 which will be added later on to calculate total knowledge score of the participant. Finally, the total knowledge score was calculated by adding all the scores. Then, the mean of the total knowledge score (3.42) was calculated and compared to the total knowledge score of each participant. Furthermore, below the mean score, the participants were considered having an inadequate level of knowledge. Otherwise, the participants were considered having an adequate level of knowledge.

The study was approved by the Nepal Health Research Council (NHRC). Permission was taken from the employer or the gatekeeper of the constructing sites for interviewing the laborers and written consents were taken from the participants after clearly explaining the purpose of the study. Furthermore, the participants were also assured about the confidentiality of the data. The data obtained were used only for the purpose of this study. Likewise, no discrimination or bias was done based on caste, sex, or religion during data collection.

The collected data was entered in EpiData version 3.1 where entry, editing, and checking of data were done. The edited data was entered in SPSS version 20 for data analysis. Descriptive statistics including frequency distribution and mean and standard deviation were used to describe the data for the sample. Cross tabulation and chi-square test were used to show the association between variables in which *p* value < 0.05 was considered the cut-off point for statistical significance.

## 3. Results

Table 1 shows that the study population consisted of 229 laborers. Among all the participants, the mean age was 30 years; majority were male (86.5%), illiterate (25.3%), married (72.5%), Adhibasi/Janajati (61.1%), Hindu (76.4%), nuclear family type (66.4%), and residing in rural areas (61.1%).

Table 2 explains the knowledge of laborers on OSH. Among the 229 participants, the minority (10.9%) heard about OSH, 38.4% heard about first aid, 1.7% heard about OSH program, and 6.6% heard about OSH training. Maximum participants (92.1%) heard about PPE and only 6.1% and 3.5% heard about safety inspection and safety meeting, respectively. It was found that more than half of the laborers (66.8%) heard about the preventive and control measures of occupational accidents and only 2.2% of the participants heard about OSH policy. Only 82.5% used PPE during their work. Table 2 also describes the level of knowledge of laborers on OSH. It was found that 62% that is more than half of the participants had inadequate knowledge and the rest had an adequate level of knowledge (38%).

Table 3 explains the association between the level of knowledge of laborers and the sociodemographic characteristic of the laborers. It shows that there was a significant association of level of knowledge of laborers with sex (*p* value < 0.001), education (*p* value < 0.001), and family type (*p* value = 0.042). Likewise, no other significant association was found between the level of knowledge of laborers and other sociodemographic characteristics since the *p* value >0.05.

Table 4 explains the prevalence of occupational accidents of the laborers within a year. It shows that the prevalence of the occupational accidents within a year was 19.7% out of which 91.1% of the participants had accidents about 1–3 times. Furthermore, most of the accidents were caused while lifting/carrying heavy objects (24.4%) and striking against fixed/stationary objects (24.4%). Moreover, 60% of injuries were mostly of hand, palm, finger, and wrist followed by 46.7% of injuries of the lower leg, ankle, sole, and toe.

Table 5 shows the association of prevalence with the level of knowledge of laborers and the use of PPE during work by the laborers. It was found that there was a significant association between the use of PPE during work by the laborers and the prevalence of occupational accidents within a year with *p* value = 0.045 and chi-square value = 4.017. Furthermore, no significant association was found between the level of knowledge of laborers and the prevalence of occupational accidents (*p* value = 0.093). Furthermore, the table shows that participants who did not have any occupational accidents within a year were 168 out of which the majority (85.1%) used PPE often during their work.

## 4. Discussion

In this study, the majority were male (86.5%) and most of the participants (49.3%) were of 21–30 years of age. 25.3% of the participants were illiterate and 24.5% had studied secondary level of education. Moreover, 72.5% were married and 34.9% of the participants had 1–5 years of experience in this field. The sociodemographic characteristic of this study was similar to the cross-sectional descriptive study on knowledge and use of Personal Protective Equipment among auto technicians in Uyo, Nigeria, where most of the participants were male (98%) and 30.5% of the participants were of age group of 22–31 years [12]. And this study was also similar to a cross-sectional study on the use of Personal Protective Equipment among Building Constructing Workers in Kampala, Uganda, where 79.2% were males and 75.3% were aged 18–30 years [13].

This study also found that all the laborers worked for 5–7 days a week and 68.6% of the laborers worked for 8–12 hours. This is against Labor Act, 2074, of Nepal where the provision related to working hours has mentioned that no workers shall be employed to work more than 8 hours a day and 48 hours a week by an employer [14].

This study found that more than half of the participant (62%) had inadequate knowledge which is similar to the report of the current situation of occupational safety and health in Nepal which reports that informal sector workers have little knowledge compared to the formal sector [15] and is in contrast to the cross-sectional descriptive study on the use of Personal Protective Equipment on constructing sites in, Nigeria, where 80.8% had good knowledge of PPE [16]. Furthermore, this study is in contrast to the study on practice related to occupational health and safety among workers of brick factories at Bhaktapur, Nepal, which found that most of the participants had knowledge about occupational health and safety [11].

Moreover, it was also found that 17.5% of participants did not use PPE and 82.5% of participants used PPE which is similar to the descriptive study on the topic effect of occupational health and safety on healthy lifestyle behaviors of workers employed in a private company in Turkey where 83.9% workers used PPE. But it is in contrast to the study on practice related to occupational health and safety among workers of brick factories at Bhaktapur, Nepal, which found that the level use of PPE was very low (86.3%) [11] and the study report on “how to reduce and eliminate” the hazardous and risky works in constructing industries of Nepal where it was found that 67% of the workers wore PPE at workplace occasionally [6]. Furthermore, the result was in contrary to the study on the use of PPE on constructing site in Nigeria which found that 81.1% did not wear PPE provided on site [16] and a cross-sectional study on the use of Personal Protective Equipment among Building Constructing Workers in Kampala, Uganda, where it was found that there was low use of PPE (15.6%) among building constructing workers [13].

Additionally, in this study, it was also found that the most used PPE were gloves (86.2%) followed by boots (79.9%) then masks (61.5%) which is similar to the study on practice related to occupational health and safety among workers of brick factories at Bhaktapur, Nepal, which found that about 54.7% of them use gloves, 25.3% use boot, and 18% use mask as Personal Protective Equipment [11]. But this study is in contrast to the cross-sectional study on the awareness of occupational hazards and use of safety measures among welders from eastern Nepal in which welding goggles/eye shields (86.7%) were the most commonly reported PPE for use since the study was done on welders only [17].

Likewise, this study also found that the occupational accidents that occurred within a year were 19.7%. This is similar to the cross-sectional study on risky work: accidents among Nepalese migrant workers in Malaysia, Qatar, and Saudi Arabia which found that almost 17% of Nepalese migrant workers had experienced work-related accidents in Qatar, Saudi Arabia, and Malaysia [18] but in contrast to the descriptive study on the topic effect of occupational health and safety on healthy lifestyle behaviors of workers employed in a private company in Turkey where, among 83.9% using PPE, only 2.1% said that they had experienced an occupational accident [19].

It was also found that mostly accidents were caused while lifting/carrying (24.4%) and striking (24.4%) against a stationary object. This is a contrast in comparison to the study on labor safety in constructing sites in India which concluded that major accidents occurred due to body injuries accounting to 44.1% in small constructing sites and 26.4% in large constructing sites [7] and the study report on “how to reduce and eliminate” the hazardous and risky works in constructing industries of Nepal which resulted in that 58% accidents were caused due to falling from a height and 10% by electric shock [6].

Also, it was found that there was a significant relation between the level of knowledge with sex (*p* value < 0.001), education (*p* value < 0.001), and family type (*p* value = 0.042) only. Since in our country, more males are preferred in the constructing work and they are given more priority to education than females, females tend to lack in number in the constructing work resulting in a low level of knowledge in comparison to males. Furthermore, the nuclear family have less number of family members than the extended and joint family type which results in limited knowledge sharing on limited fields. And it also resulted in that there was a significant association between the use of PPE and occupational accidents that occurred within a year (*p* value = 0.045) which shows that, among 168 participants who did not have accidents within a year, 143 of them used PPE and also, among 171 participants who used PPE, more than half (143) of the participants did not have any occupational accidents within a year. This can explain that the use of PPE can minimize the occurrence of occupational accidents. This is similar to the cross-sectional study on the awareness of occupational hazards and the use of safety measures among welders from eastern Nepal which showed that education was significantly associated with the use of PPE [17] but was in contrast to the cross-sectional descriptive study on knowledge and the use of Personal Protective Equipment among auto technicians in Uyo, Nigeria, where there was no significant association of level of knowledge and education [12] and to the cross-sectional study on the use of Personal Protective Equipment among Building Constructing Workers in Kampala, Uganda, where there was no statistical significant association of level of education to the use of PPE [13].

Furthermore, it was observed that the situation of the constructing site was very risky since there was a presence of exposure of laborers to hazardous areas and machineries, harmful chemicals, dust, and repetitive movements. This is in relation to the statement that constructing laborers are exposed to a wide variety of health hazards on the job mentioned at the General Survey on the occupational safety and health instruments concerning the promotional framework, constructing, mines, and agriculture done by International Labor Conference, 2017 [20].

Although some participants had difficulty in recalling the number of accidents within 12 months (recall period), this paper will still help the researchers to know about the current situation of OSH knowledge and practices of laborers.

## 5. Conclusion

This study will help the employer and policymaker to build effective policies for improving the working environment for the laborers and implement the required awareness and training programs for the improvement in the knowledge and practice of OSH of the laborers. Moreover, further study can be done to identify the gap between the knowledge and use of PPE and factors that affect the utilization of PPE of the laborers in constructing sites.

## Figures and Tables

**Table 1:** Sociodemographic characteristics of the participants (*n* = 229).

Characteristics	*n* (%)
Age, mean (SD)	30 ± 9.705 years
Gender	
Male	198 (86.5)
Female	31 (13.5)

Education	
Illiterate	58 (25.3)
Primary	47 (20.5)
Lower secondary	49 (21.4)
Secondary	56 (24.5)
Higher secondary and above	19 (8.3)

Marital status	
Married	166 (72.5)
Single	61 (26.6)
Divorced	2 (0.9)

Ethnicity	
Brahmin/Chhetri	25 (10.9)
Madhesi	28 (12.2)
Dalits	21 (9.2)
Adhibasi/Janajati	140 (61.1)
Muslim	12 (6.6)

Religion	
Hindu	175 (76.4)
Non-Hindu	54 (23.6)

Family type	
Nuclear	152 (66.4)
Joint	65 (28.4)
Extended	12 (5.2)

Place of permanent residence	
Rural	140 (61.1)
Urban	89 (38.9)

**Table 2:** Knowledge of laborers on OSH and related variables (*n* = 229).

Variables	*n* (%)
Heard about OSH	
Yes	25 (10.9)
No	204 (89.1)
Heard about first aid	
Yes	88 (38.4)
No	141 (61.6)
Heard about OSH program	
Yes	4 (1.7)
No	225 (98.3)
Heard about OHS training	
Yes	15 (6.6)
No	214 (93.4)
Heard about PPE	
Yes	211 (92.1)
No	18 (7.9)
Use PPE during work (*n* = 211)	
Yes	174 (82.5)
No	37 (17.5)
Heard about safety inspection	
Yes	14 (6.1)
No	215 (93.9)
Heard about safety meeting	
Yes	8 (3.5)
No	221 (96.5)
Heard about preventive and control measures of occupational accidents	
Yes	153 (66.8)
No	76 (33.2)
Heard about OSH policy	
Yes	5 (2.2)
No	224 (97.8)

Level of knowledge	*n* (%)
Inadequate knowledge	142 (62)
Adequate knowledge	87 (38)
Total	100

Below mean 3.42 = inadequate knowledge, above mean 3.42 = adequate knowledge.

**Table 3:** Association between the level of knowledge of laborers and soci-demographic variables (*n* = 229).

Variables	Level of knowledge		Total *N* = 229	*p* value	*χ* ^2^ value
	Inadequate *n* = 142 *n* (%)	Adequate *n* = 87 *n* (%)			
Sex					
Male	114 (57.6)	84 (42.4)	198	<0.001^*∗*^	12.201
Female	28 (90.3)	3 (9.7)	31		

Age					
20 years or below	23 (71.9)	9 (28.1)	32	0.107	7.621
21–30 years	63 (55.8)	50 (44.2)	113		
31–40 years	30 (58.8)	21 (41.2)	51		
41–50 years	18 (81.8)	4 (18.2)	22		
Above 50 years	8 (72.7)	3 (27.3)	11		

Education					
Illiterate	50 (86.2)	8 (13.8)	58	<0.001^*∗*^	30.786
Primary	31 (66)	16 (34)	47		
Lower secondary	30 (61.2)	19 (38.8)	49		
Secondary	26 (46.4)	30 (53.6)	56		
Higher secondary and above	5 (26.3)	14 (73.7)	19		

Marital status					
Married	100 (60.2)	66 (39.8)	166	0.412	1.775
Single	40 (65.6)	21 (34.4)	61		
Divorced	2 (100)	0	2		

Family type					
Nuclear	103 (67.8)	49 (32.2)	152	0.042^*∗*^	6.357
Joint	33 (50.8)	32 (49.2)	65		
Extended	6 (50)	6 (50)	12		

Ethnicity					
Brahmin/Chhetri	15 (60)	10 (40)	25	0.316	4.730
Madhesi	13 (46.4)	15 (53.6)	28		
Dalits	15 (71.4)	6 (28.6)	21		
Adhibasi/Janajati	91 (65)	49 (35)	140		
Muslim	8 (53.3)	7 (46.7)	15		

Religion					
Hindu	105 (60)	70 (40)	175	0.251	5.371
Buddhism	22 (81.5)	5 (18.5)	27		
Islam	8 (53.3)	7 (46.7)	15		
Christianity	5 (62.5)	3 (37.5)	8		
Other	2 (50)	2 (50)	4		

Place of permanent residence					
Rural	84 (60)	56 (40)	140	0.432	0.617
Urban	58 (65.2)	31 (34.8)	89		

^*∗*^ *p* value is less than 0.05.

**Table 4:** Prevalence of occupational accidents within a year (*n* = 229).

Variables	Frequency	
Occupational accidents within a year		
Yes	45 (19.7)	
No	184 (80.3)	

If yes, occupational accidents occurred (*n* = 45)		
1–3 times	41 (91.1)	
4–8 times	4 (8.9)	

Cause of accidents^*∗*^ (*n* = 45)		
Slipping, tripping, and falling on the same level	8 (17.8)	
Injured while lifting/carrying objects	11 (24.4)	
Striking against fixed/stationary objects	11 (24.4)	
Injured by hand tools	7 (15.6)	
Injured by falling objects	7 (15.6)	
Other causes	4 (8.9)	

Injured body parts^*∗*^ (*n* = 45)		
Hand, palm, finger, wrist	27 (60)	
Lower leg, ankle, sole, toe	21 (46.7)	
Skull, scalp, neck	2 (4.4)	
Chest and abdomen	1 (2.2)	

^*∗*^Multiple responses.

**Table 5:** Association between the prevalence of occupational accidents with the level of knowledge of laborers on OSH and the use of PPE (*n* = 229).

Variables	Occupational accidents within a year		*p* value	*χ* ^2^ value
	Yes *n* (%)	No *n* (%)		
Level of knowledge (*n* = 229)				
Low level of knowledge on OHS	23 (51.1)	119 (64.7)	0.093	2.823
Adequate knowledge on OHS	22 (48.9)	65 (35.3)		
Total	45 (100)	184 (100)		
Use of PPE (*n* = 211)				
Yes	31 (72.1)	143 (85.1)	0.045^*∗*^	4.017
No	12 (27.9)	25 (14.9)		
Total	43 (100)	168 (100)		

^*∗*^ *p* value is less than 0.05.

## Data Availability

The data used in the study will not be shared publicly. The datasets and materials used and/or analyzed and the software application or custom codes used in the research can be made available from the corresponding author on reasonable request.
